# Design of a Board-Level Integrated Multi-Channel Radio Frequency Source for the Transportable ^40^Ca^+^ Ion Optical Clock

**DOI:** 10.3390/s25041044

**Published:** 2025-02-10

**Authors:** Bin Wang, Yuanhang Yang, Huaqing Zhang, Ruming Hu, Haicen Mao, Yao Huang, Kelin Gao, Hua Guan

**Affiliations:** 1 Key Laboratory of Time Reference and Applications, Innovation Academy for Precision Measurement Science and Technology, Chinese Academy of Sciences, Wuhan 430071, China; wangbin192@mails.ucas.ac.cn (B.W.); hqzhang@wipm.ac.cn (H.Z.); huruming@apm.ac.cn (R.H.); yaohuang@wipm.ac.cn (Y.H.); klgao@wipm.ac.cn (K.G.); 2 University of Chinese Academy of Sciences, Beijing 100049, China; 3 Huazhong Institute of Electro-Optics, Wuhan National Laboratory for Optoelectronics, Wuhan 430223, China; yyh971125@163.com (Y.Y.); 2023810013@hust.edu.cn (H.M.); 4 Wuhan Institute of Quantum Technology, Wuhan 430206, China

**Keywords:** transportable ^40^Ca^+^ ion optical clock, signal source, direct digital synthesis, power amplifier

## Abstract

As one of the most precise timekeeping instruments ever developed, the optical clock will be used as the measuring equipment for the next generation of second definition. The demand for the miniaturization of optical clocks is progressively urgent. In this paper, a multi-channel radio frequency (RF) module with a 20% volume of the commercial module is designed and implemented for the transportable ^40^Ca^+^ ion optical clock. Based on the double-crystal oscillator interlocking technique, a 1 GHz low-phase noise reference source is developed for direct digital synthesis. Through the simulation and optimization of the signal link design, the frequency range of the low phase-noise RF signal can reach 0–400 MHz with a 4 μHz resolution. Through two-stage power amplifying with different kinds of filters, it can achieve an output power of up to +33 dBm (2 W) at 100 MHz with a 25 dB phase noise lower than the commercial module at 1 Hz, and its third harmonic suppression ratio has been reduced by more than 20 dB at the frequency point of 300 MHz. This multi-channel RF module is used for the power stability and timing control test of a 729 nm clock laser to meet the requirements of the transportable ^40^Ca^+^ optical clock. Additionally, this module can also be applied to other quantum systems such as the quantum absolute gravimeter, quantum gyroscopes, and quantum computers.

## 1. Introduction

With the rapid development of laser cooling [1,2,3,4,5], the ultra-narrow linewidth laser [6,7,8,9], and the femtosecond optical frequency comb [10,11], optical clocks play an important role in the measurement of basic physical constants [12], the verification of general relativity [13,14], gravitational wave measurement [15,16,17], and other precise measurement physics [18,19]. In recent years, with the increasingly urgent demand for the next generation of second definition [20] and the measurement of the elevation difference [21,22], the miniaturization research of the key sensing components of the optical clock has been a widespread concern [23,24]. The working operation of optical clocks involves complex laser modulation technology, and radio frequency (RF) modules are indispensable for driving or modulating the acousto-optic modulators (AOMs) and electro-optic modulators (EOMs) in this process. Therefore, RF modules stand as a key component of the optical clock, and their miniaturization research is of great significance in meeting the requirements of transportable optical clocks.

At present, the RF module in laboratory-based clock systems is mainly provided in two ways [25,26,27,28]: one is the combination of a commercial signal source and a commercial power amplifier module, which is used to provide an RF driver. For example, in the ^40^Ca^+^ optical clock, the Innovation Academy for Precision Measurement Science and Technology (APM), Chinese Academy of Sciences (CAS) uses 5183B and direct digital synthesis (DDS) modules as the signal source [29]. Additionally, they use a commercial broadband power amplifier to amplify the signal source. Another way is the combination of a self-developed signal source and a commercial power amplifier module. For example, in the ^9^Be^+^ ion optical clock of the University of Science and Technology of China, the self-developed 4-channel 2.8 Gsps@14-bit arbitrary waveform generator and 16-channel 1 Gsps@14-bit DDS module are used as a signal source and then pass a commercial power amplifier as the RF driver, which can improve the integration of the system [30].

To further improve the integration of the system, we use a self-developed signal source and power amplifier as the RF driving module. The integration of the module is improved by mechanical–electrical joint design, which solves the size problem for integration in the transportable ^40^Ca^+^ optical clock system. Based on the double-crystal oscillator interlocking technique, its output frequency range can reach 0–400 MHz with a 4 μHz resolution, and the output power is up to +33 dBm. Finally, this module is tested by the 729 nm clock laser power stabilization and timing control, which can meet the requirement of the transportable ^40^Ca^+^ optical clock system.

The rest of this paper is as follows: Section 2 introduces the design method and key technologies of the integrated RF module and introduces the performance test results of the module; Section 3 introduces the experimental composition of the RF module applied to the ^40^Ca^+^ optical clock and the test results of power stability and timing control of the 729 nm laser; and Section 4 outlines the conclusion.

## 2. Design of Integrated RF Source

The integrated RF source consists of two parts: an RF signal source and a power amplifier, which is usually used to drive or modulate the AOMs and EOMs in the laser path of a transportable ^40^Ca^+^ optical clock system. Its frequency and power can be changed in real time according to the time sequence requirements in the optical clock experiment process.

### 2.1. RF Signal Source

At present, the design methods of RF signal sources mainly include direct analog frequency synthesis (DAS), a phase-locked loop (PLL) [31,32,33], and DDS [34,35]. In this design, the advantages of PLL and DDS are combined, and the mixed-frequency synthesis technology of PLL + DDS is adopted. The phase noise of the DDS output signal depends on the stability of the reference clock. To reduce the influence of the reference clock’s phase noise, firstly, double-crystal oscillator interlocking technology is adopted to improve the near-end phase noise of the 100 MHz crystal oscillator through a 10 MHz crystal oscillator of PLL. Secondly, the 100 MHz signal is multiplied to 1 GHz by the second PLL as the input clock of DDS, and then any frequency within 400 MHz is generated by DDS frequency division. The output frequency $f_{out}$ of the DDS can be expressed as

(1) $$f_{out}=\frac{FTW}{2^{N}}\cdot f_{c}$$

where *FTW* is the frequency control words, *N* is the number of bits in the DDS chip, and $f_{c}$ is the reference frequency. According to Nyquist’s sampling theorem, a unique analog signal can be obtained only when the sampling signal frequency is more than twice the signal frequency; theoretically, the maximum output frequency of the DDS is half of its input frequency. However, due to the digital characteristics and process effects of the DDS chip, the actual maximum output frequency often cannot reach $f_{c}∕2$. Typically, the output frequency of the DDS chip is one-third of the reference clock frequency, which can reach 40% with process improvements.

**Figure 1:**
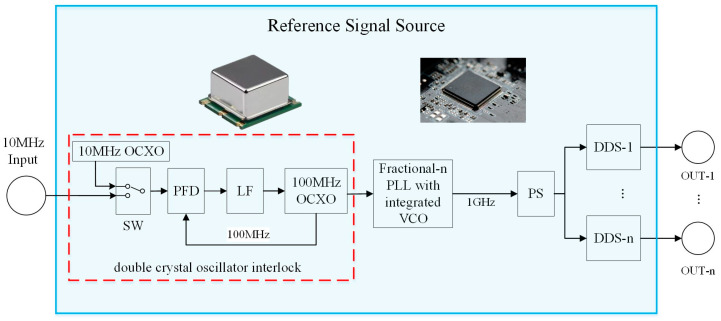
System block diagram of integrated RF source. The red dotted box shows the composition of the double crystal oscillator interlock circuit. The abbreviations in the figure are as follows: OCXO: oven-controlled crystal oscillator; SW: signal selector switch; PFD: phase-frequency detector; LF: loop filter; VCO: voltage-controlled oscillator; PS: power splitter.

The block diagram of the designed RF signal source is shown in Figure 1. It is mainly composed of a signal selector switch (SW, SN74LVC1G3157), a phase-frequency detector (PFD, HMC4069), a loop filter (LF), an oven-controlled crystal oscillator (OCXO, O22B-O429-100.00 MHz), and Fractional-n PLL with an integrated voltage-controlled oscillator (VCO, HMC834). This system is designed to convert the 10MHz input signal into a 1 GHz output signal. The DDS circuit is composed of a power splitter (PS, ADP-2-1W), DDS (AD9912), a balun circuit, etc. It multiplies and divides the 1 GHz signal from the reference clock to output the frequencies needed by the system. The 100 MHz signal interlocked by the double-crystal oscillator is frequently multiplied to 1 GHz as the input clock of DDS. The phase noise brought by using the external signal source is much lower than that of the PLL frequency multiplier integrated into DDS, which can effectively improve the phase noise of the DDS output signal.

**Figure 2:**
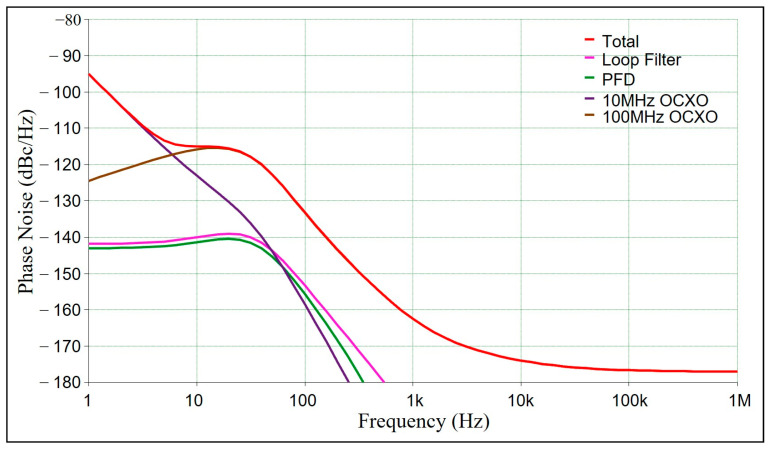
Phase noise simulation diagram of 100 MHz at 1 Hz–1 MHz frequency offset. The diagram shows that phase noise in the loop bandwidth is determined by 10 MHz OCXO, and the phase noise outside the loop bandwidth is determined by the 100 MHz OCXO.

For a signal of 10 MHz, the switch can be used to select the input reference signal from an external hydrogen clock or a crystal oscillator. Typically, the types of crystal oscillators include ordinary crystal oscillators, temperature-compensated crystal oscillators, voltage-controlled crystal oscillators, or oven-controlled crystal oscillators. Among these, oven-controlled crystal oscillators have the highest frequency stability with an order of 10^−9^, minimal environmental temperature influence, and the best phase noise performance, making them suitable as reference signal sources for RF modules. In some experiments, the dual crystal oscillator interlocking technology, using a phase-locked loop with a 10 MHz crystal oscillator to improve the near-end phase noise output of a 100 MHz crystal oscillator, is employed to enhance system stability. ADIsimPLL is a phase-locked loop simulation software provided by Analog Devices, Inc. (ADI), which can simulate quickly according to the chip model directly. The double-crystal oscillator interlock circuit is shown in the red dotted-line box in Figure 1. Using ADIsimPLL to simulate the phase noise, its signal is 100 MHz and the frequency offset is 1 Hz–1 MHz. The loop bandwidth is set to 30 Hz and the phase margin is set to 48 degrees. The result is shown in Figure 2, where the phase noise at a 1 Hz frequency offset is −95 dBc/Hz.

The AD9912 has a maximum reference clock of 1 GHz, a maximum output frequency of 400 MHz, an integrated 14-bit DAC, a 48-bit frequency tuning word, and a frequency resolution of 4 μHz. The AD9912 outputs a differential signal, which needs to be converted into a single-ended signal before it can enter the filtering and amplification circuit. A balun circuit is constructed using an RF transformer to match the balanced port with the single-ended port.

### 2.2. Power Amplification Circuit

The output signal bandwidth of the RF signal source is 400 MHz, and the power is about 0 dBm. The frequency requirement of the ^40^Ca^+^ optical clock for the RF input signal is within 250 MHz. To drive the AOM, the signal power needs to be amplified to nearly +33 dBm (2 W). The difficulty of power amplification link design is to amplify the effective signal and reduce the noise as much as possible. The noise factor *F* and noise figure *NF* are commonly used as metrics for the degradation of the signal-to-noise ratio (SNR) caused by the RF circuit. The noise factor is defined as the ratio of the input *SNR* to the output *SNR*, and the noise figure is defined as the logarithmic value of the noise factor.

(2) $$F=\frac{Input\;Port\;SNR}{Output\;Port\;SNR}$$

(3) NF=logF

**Figure 3:**
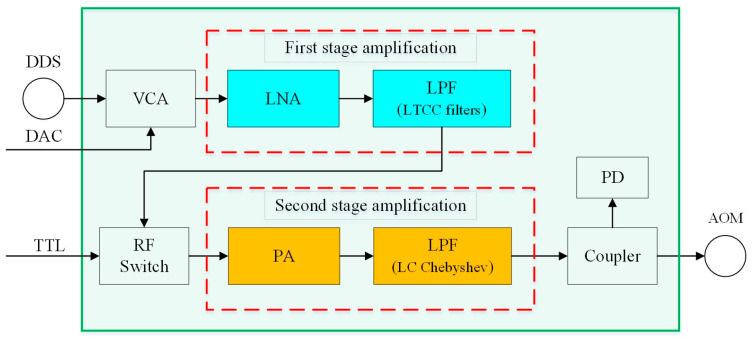
Schematic diagram of power amplifier circuit. The red dotted boxes show the composition of the first stage and the second stage amplification circuit. The abbreviations and device models in the figure are as follows: VCA: voltage control attenuator, HMC346; LNA: low-noise amplifier, LTC6433; LPF: low-pass filter; LTCC filters: LFCN–400+; RF switch: HMC1118; PA: power amplifier, MW6S004NT1; PD: power detector, ADL5906; DAC: digital–analog converter; TTL: transistor–transistor logic.

For cascaded RF components, the noise figure of each stage can be calculated using the following formula:

(4) $$F=F_{1}+\frac{F_{2}-1}{G_{1}}+\frac{F_{3}-1}{G_{1}\times G_{2}}+\ldots +\frac{F_{n}-1}{G_{1}\times G_{2}\times \ldots \times G_{n-1}}$$

where $F_{n}$ represents the noise factor of each stage of RF components and $G_{n}$ represents the gain of each stage. When the gain of the power amplifier (PA) is much greater than the noise factor, the overall noise of the power amplifier link is mainly determined by the first-stage one. To reduce the noise introduced by the power amplifier circuit, a two-stage power amplifier is used. The noise generated by the power amplifier is proportional to the amplification bandwidth. The larger the bandwidth, the more noise is introduced. Therefore, the first stage uses a narrow-band low-noise amplifier to amplify the DDS signal, and the second stage uses laterally diffused metal oxide semiconductor (LDMOS) tubes to amplify the power. Because the PA amplifies the baseband signal as well as the harmonic and clutter signal, especially the second harmonic, it is necessary to add a low-pass filter after each stage of PA to filter out the harmonics, which is an essential part of the power amplifier link. The low-temperature co-fired ceramic (LTCC) filter has the advantages of small volume, small insertion loss, and good gain flatness. Therefore, an LTCC low-pass filter is used after the first stage of the low-noise amplifier. The filter model selected is LFCN–400+, its pass frequency is DC–400 MHz, and its 3 dB corner frequency is 560 MHz. After the second-stage power amplifier (PA), it is necessary to choose a filter with a narrow passband bandwidth, small volume, and fast transition band attenuation, so an LC Chebyshev low-pass filter with a 3 dB corner frequency of 500 MHz is selected. The structure of the designed power amplifier circuits is shown in Figure 3.

**Figure 4:**
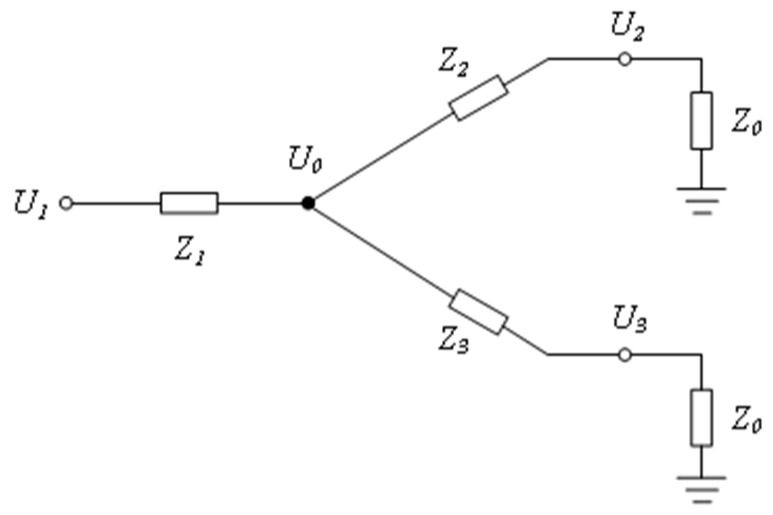
Schematic diagram of resistive coupler/power divider.

To detect the output power of the amplification link in real time, a power detection circuit is designed, which converts the power information of the RF signal into voltage and transmits it to the upper computer through an analog-to-digital converter for real-time RF power monitoring. The input power of the RF detector is in the range of −60 to 2 dBm. Since the maximum output power of the power amplifier is above 33 dBm, a coupler is needed to divert a small portion of the RF signal for detection purposes. To save space, the lumped parameter resistive coupler is directly used to separate a small part of the RF power signal for power monitoring, as shown in Figure 4.

In the diagram, $U_{1}$ represents the input port, $U_{2}$ is the output port, and $U_{3}$ is the coupling port. $Z_{0}$ denotes the characteristic impedance of the circuit, which is designed to be 50 Ω in this setup. The values of $Z_{1}$, $Z_{2}$, and $Z_{3}$ can be calculated by setting a proportionality coefficient k as follows:

(5) $$\left.\begin{array}{c} P_{1}=P_{2}+P_{3}\\ {U_{2}}^{2}=k{U_{3}}^{2}\\ U_{0}=\frac{\left(Z_{2}+Z_{0}\right)//\left(Z_{3}+Z_{0}\right)}{\left(Z_{2}+Z_{0}\right)//\left(Z_{3}+Z_{0}\right)+Z_{1}}U_{1}\\ \frac{U_{1}-U_{0}}{Z_{1}}=\frac{U_{0}-U_{2}}{Z_{2}}+\frac{U_{0}-U_{1}}{Z_{3}}\\ \left(Z_{2}+Z_{0}\right)//\left(Z_{3}+Z_{0}\right)+Z_{1}=Z_{0} \end{array}\right\}$$

In the power detection circuit, the relevant parameters are set as follows: $Z_{1}=Z_{2}=1\;\Omega$, $Z_{3}=1200\;\Omega$, k =1000.

### 2.3. Design Result

**Figure 5:**
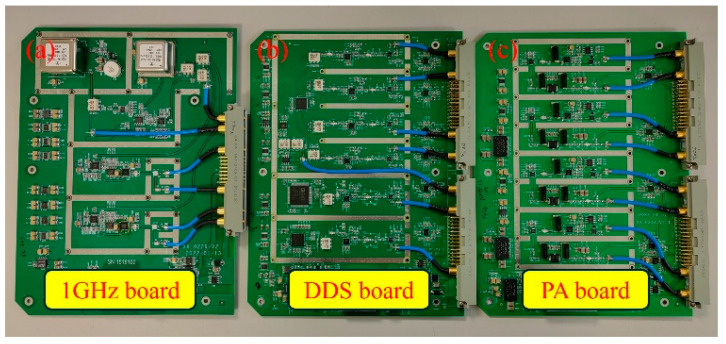
Photograph of the PCB boards. (**a**) 1 GHz board, (**b**) DDS board, and (**c**) PA board.

Mechanical–electrical joint design is adopted to improve the module’s integration. Three kinds of circuit boards are developed. A photograph of the PCB boards is shown in Figure 5. The size of the 1 GHz board is 170 mm × 230 mm × 40 mm, the DDS board is 170 mm × 230 mm × 20mm, and the PA board is 170 mm × 230 mm × 20 mm. The volume of the self-made circuit board is about 20% of that of a commercial one, which greatly reduces the occupied space and improves system integration.

We used the FSWP26 phase noise analyzer from Rohde & Schwarz for the test experiments. The FSWP26 phase noise analyzer includes both spectrum analysis and phase noise analysis modes, allowing the signal’s spectrum and phase noise to be tested through the same port.

**Figure 6:**
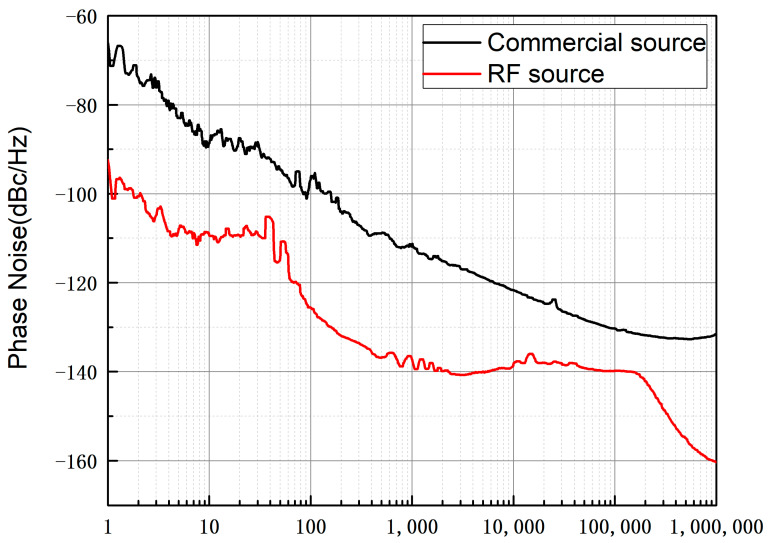
Output signal phase noise test chart. The phase noise of the RF module is better than that of the commercial signal source by more than 25 dB at the frequency point of 1 Hz.

The phase noise of the RF module’s DDS board output signal was tested against the 100 MHz signal output from a commercial signal source (ARTIQ DDS Urukul card). The reference clock for both sources was a 10 MHz signal from the same hydrogen maser in the laboratory. The phase noise analyzer was set to a resolution bandwidth of 3%, a cross-correlation factor of 10, and a frequency offset range of 1 Hz to 1 MHz. The phase noise test plot is shown in Figure 6. The main reason for the better performance of the phase noise of the RF module is that the integrated VCO phase-locked loop is used to double the frequency of the crystal oscillator as the DDS reference signal, while the commercial signal source uses the integrated frequency multiplier in the DDS chip as the reference signal, which will lead to the phase noise deterioration of 10–20 dB.

**Figure 7:**
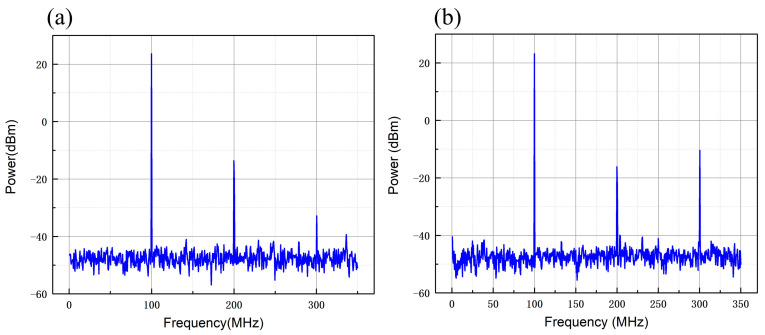
Output signal spectrum test chart. (**a**) PA board 100 MHz carrier spectrum diagram; (**b**) commercial source 100 MHz carrier spectrum diagram. The third harmonic rejection ratio of the PA board is better than commercial source by more than 20 dB.

The output signal of the PA board and the commercial source (ZHL-1-2W-S+) are tested, respectively. We set the RF module’s DDS board output signal to 100 MHz, the resolution bandwidth of the phase noise meter to 100 kHz, the RF bandwidth to 100 kHz, the center frequency to 100 MHz, and the test frequency range to 300 MHz. The spectrum test diagram is shown in Figure 7. The test results show that when the output frequency is 100 MHz and the power amplification is 33 dBm (2 W), the third harmonic rejection ratio of the PA board is better than the commercial source by more than 20 dB.

**Figure 8:**
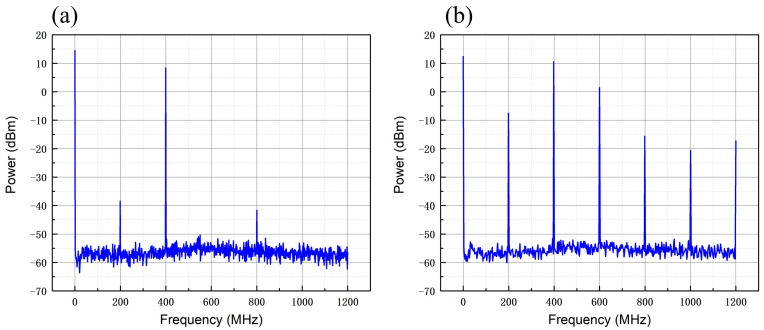
Output signal spectrum test chart. (**a**) PA board 400 MHz carrier spectrum diagram; (**b**) commercial source 400 MHz carrier spectrum diagram.

We then set the RF module’s DDS board output signal to 400 MHz, the resolution bandwidth of the phase noise meter to 100 kHz, the RF bandwidth to 100 kHz, the center frequency to 600 MHz, and the test frequency range to 1200 MHz. The spectrum test diagram is shown in Figure 8. The test results show that when the output frequency is 400 MHz and the power amplification is 33 dBm (2 W), the PA board is superior to the commercial source in suppressing higher harmonics.

**Figure 9:**
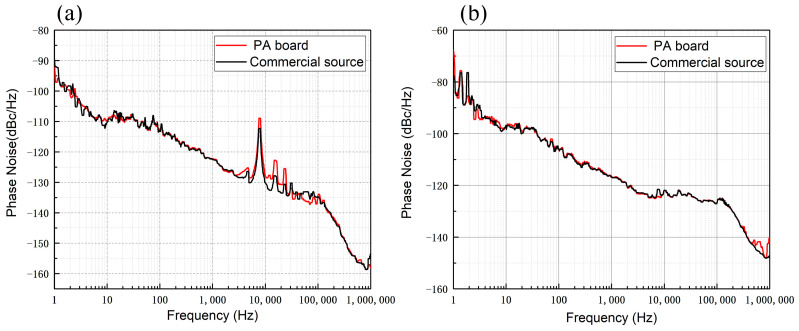
PA board and commercial module output phase noise test chart. (**a**) The phase noise comparison between PA board and commercial source when the output signal is 100 MHz; (**b**) the phase noise comparison between PA board and commercial source when the output signal is 400 MHz. At the output frequency of 100 MHz, the phase noise is better than that at the output frequency of 400 MHz at the frequency point of 1 Hz.

The phase noise of the PA board and commercial module (ZHL-1-2W-S+) with output signals of 100 MHz and 400 MHz are tested, respectively. The phase noise analyzer was set with a resolution bandwidth of 3%, a cross-correlation factor of 10, and a frequency offset range of 1 Hz to 1 MHz. The phase noise test plot is shown in Figure 9. The phase noise of the PA board and the commercial module is basically the same at 100 MHz and 400 MHz. When the output frequency of the PA board and the commercial module is 100 MHz, the phase noise is better than that of 20 dB at the frequency point of 1 Hz when the output frequency is 400 MHz.

**Figure 10:**
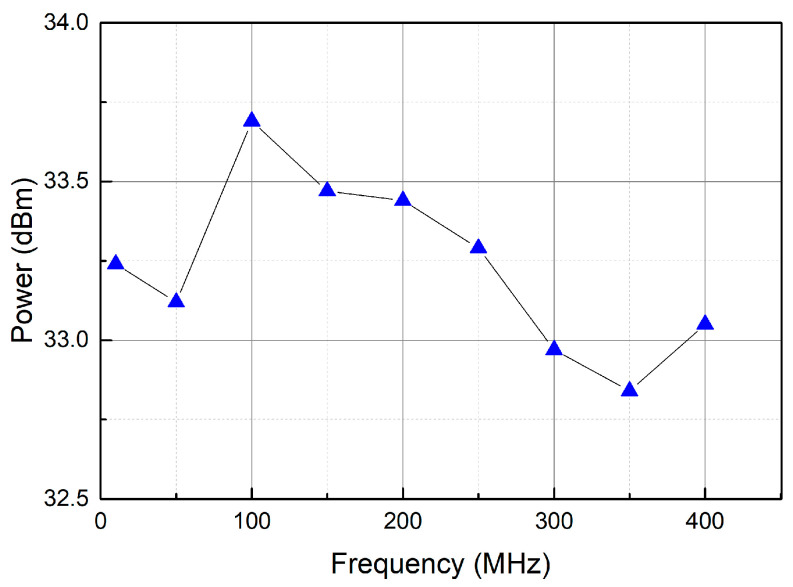
Frequency versus output power of the PA board in the bandwidth range of 400 MHz.

We then tested the output power of the PA board at different frequencies. We set the RF module’s DDS board output signal to 10–400 MHz and tested the output power of the PA board at different output frequencies. The relationship between the frequency and output power is shown in Figure 10. In the bandwidth range of 400 MHz, the output power fluctuation of the PA board is less than 1 dB.

## 3. Experimental Results and Analysis

In the laser system of a ^40^Ca^+^ optical clock, the laser at 729 nm, which is the clock transition, has the highest requirement. The laser entering the ultra-stable cavity should be stabilized in laser power to reduce the change in thermal noise of the cavity mirror caused by the laser frequency jitter, and the laser entering the ion trap should be controlled in time sequence.

**Figure 11:**
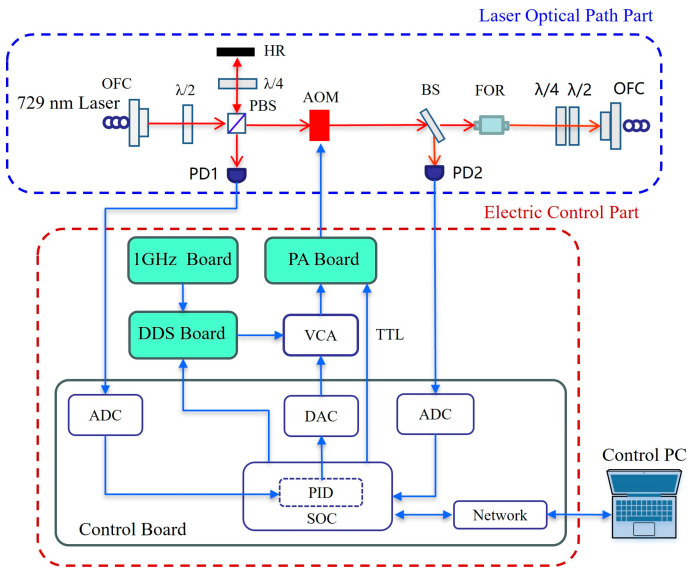
Functional block diagram of the experimental device. The abbreviations in the figure are as follows: OFC: optical fiber coupler; λ/2: half-wave plate; λ/4: quarter-wave plate; PBS: polarizing beam splitter; BS: beam splitter; PD: photo detector; PID: proportional–integral–derivative circuit.

Figure 11 shows the functional block diagram of the experimental device. The whole experimental device consists of a laser optical path part and an electric control part, where the light path part consists of a quarter-wave plate (λ/4), a polarizing beam splitter (PBS), AOM (SGT80-729-1TA-B30), a faraday rotator (FOR, FR730-3-1.5W), etc. The electronic control part consists of a control board, a 1 GHz board, a DDS board, a PA board, etc. The control board uses a system on chip (SOC, XC7Z045) as the control chip, connects to the DDS board through the input/output port to configure the AD9912, collects the output signal of the photo detector (PD, S5972) through an analog–digital converter (ADC, AD9266), controls the voltage control attenuator (VCA, ZX73-2500M-S+) through the digital–analog converter (DAC, AD9747), controls the RF switch on the PA board through transistor–transistor logic (TTL), and the computer software connects to the control board through the network port to configure various parameters in the experiment.

### 3.1. 729 nm Clock Laser Power Stability Test

**Figure 12:**
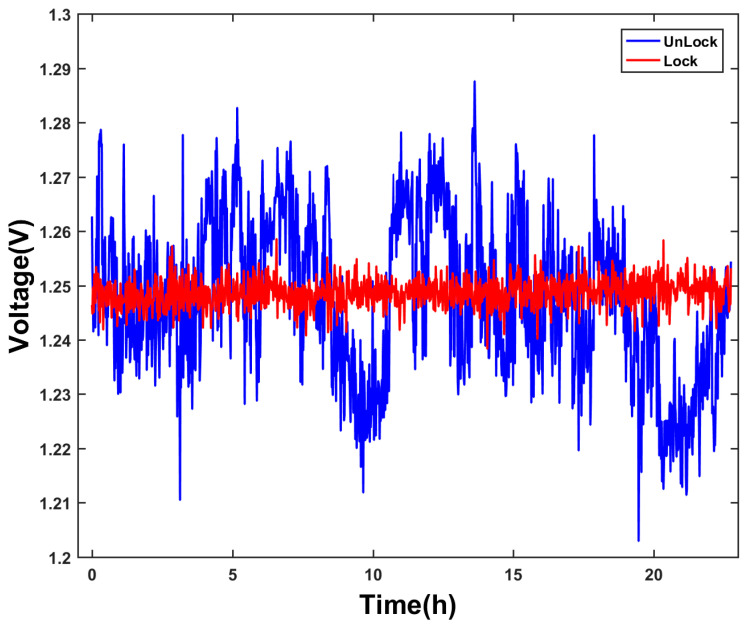
Comparison chart before and after optical power stabilization. According to the optical power data converted from the sampling voltage, the standard deviation of optical power fluctuation decreased by 5 times after the clock laser power stabilization was turned on.

The system block diagram is shown in Figure 11. In the 729 nm laser power stabilization experiment, the control board was equipped with a DDS board to output the RF signal, which was driven by VCA and the PA board. In the feedback loop, the reflected light in PBS was used as the input source of the feedback part of the power stabilization circuit, and PD1 was used to convert the optical power signal into a voltage signal. The voltage signal output by PD1 was collected by ADC on the control board and converted into a digital signal. After PID feedback calculation, it was controlled by DAC on the control board. The ADC on the main board is used to collect the voltage signal output by PD2, and the laser power change in the 729 nm clock is obtained through conversion. The ^40^Ca^+^ optical clock requires the input power fluctuation of the 729 nm laser to be less than 5%. Figure 12 shows the laser power curve before and after the clock the laser power is stably locked. In the experiment, the input power of the 729 nm laser is 180 μW and the power fluctuation range before power stabilization is 20 μW, for which power fluctuation is 11%. The fluctuation range of power after stabilization is 2.88 μW, and its power fluctuation is 1.6%, which meets the requirements of the ^40^Ca^+^ optical clock.

### 3.2. 729 nm Clock Laser Timing Control Test

**Figure 13:**
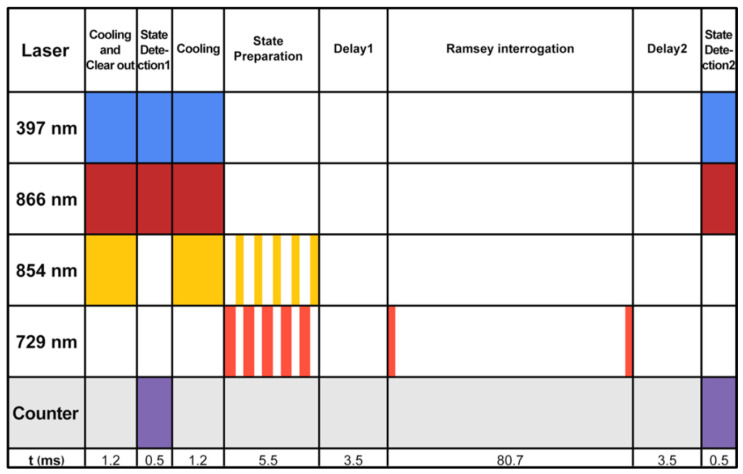
Time sequence diagram of optical clock operation. Color blocks and white blocks represent the opening and closing of the corresponding beams, respectively, and Delay1 and Delay2 are the waiting times for all lasers except the detection laser to close or open the mechanical shutter.

The operation of a ^40^Ca^+^ optical clock is mainly the process of preparing and manipulating calcium ions by controlling the laser, magnetic field, and RF field using a control system. The operation time sequence can be divided into four stages: Doppler cooling, state preparation, clock transition detection, and quantum state detection. Figure 13 shows the Ramsey detection scheme timing diagram of the current laboratory calcium ion light clock [36].

**Figure 14:**
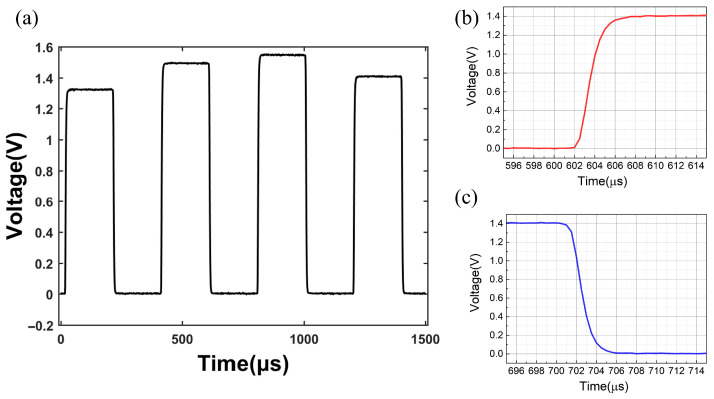
Sequential operation test chart. (**a**) is the laser power passing through the AOM can be changed by DDS, and the laser can be turned on and off. (**b**) Enlarged diagram of rising edge; (**c**) enlarged diagram of falling edge. The response time of the AOM is less than 6 μs.

The test flow of the running time sequence is as follows: First, the control board controls the DDS module to generate the RF signal, which is added to AOM through the RF switch on the PA board and then generates a TTL logic signal to control the RF switch. Finally, the voltage signal output by PD2 is collected by ADC on the control board, and the response time of AOM can be measured. In the ^40^Ca^+^ optical clock, the time required for responding to AOM is less than 100 μs. The timing test is shown in Figure 14. The laser power passing through the AOM can be changed by DDS, and the laser can be turned on and off. The response time of the AOM is less than 6 μs, which meets the requirements of timing control of the ^40^Ca^+^ optical clock.

## 4. Conclusions

We have developed a miniaturized multi-channel RF module that can provide a signal source and power amplifier at the same time with a 170 mm × 230 mm × 80 mm size. Using the PLL + DDS two-stage power-amplification technique and choosing different kinds of filters for the appropriate frequency, the output of this module within 400 MHz can reach a very low phase noise with a resolution of 4 μHz, and the output power of this module is up to +33 dBm (2 W) 100 MHz. Compared to the commercial module, the phase noise is reduced by more than 25 dB@1 Hz, and the third harmonic suppression ratio at the frequency point of 300 MHz is reduced by more than 20 dB with a volume of only 20% of that of the commercial module. Then, this module was successfully used to stabilize the 729 nm laser power in a ^40^Ca^+^ optical clock. The standard deviation of the optical power fluctuation can be decreased by 5 times with an on-and-off response time of within 6 μs. The timing control experiment results of this module show that this module can satisfy the RF requirements of the ^40^Ca^+^ optical clock system and is of great significance for the transportable ^40^Ca^+^ optical clock. Nevertheless, this module can also be extended to other quantum systems such as quantum absolute gravimeters, quantum gyro, and quantum computers.

## Data Availability

Relevant data are available upon reasonable request to the corresponding author.
